# Crystal structure and magnetic study of the complex salt [RuCp(PTA)_2_–μ-CN-1κ*C*:2κ*N*–RuCp(PTA)_2_][Re(NO)Br_4_(EtOH)_0.5_(MeOH)_0.5_]

**DOI:** 10.1107/S2056989021006381

**Published:** 2021-06-30

**Authors:** Mario Pacheco, Natalia Alvarez, Alicia Cuevas, Antonio Romerosa, Francesc Lloret, Carlos Kremer

**Affiliations:** a Area Quimica Inorganica, Facultad de Quimica, Universidad de la República, 11800, Montevideo, Uruguay; b Area de Quimica Inorganica-CIESOL, Universidad de Almeria, 04120 Almeria, Spain; c Instituto de Ciencia Molecular, Universidad de Valencia, C/ Catedratico Jose, Beltran 2, 46980 Paterna, Valencia, Spain

**Keywords:** X-ray structure, ruthenium(II), rhenium(II), PTA, magnetism, crystal structure

## Abstract

A new Ru^II^–Re^II^ complex salt, μ-cyanido-κ^2^ *C*:*N*-bis­[(η^5^-cyclo­penta­dien­yl)bis(3,5,7-tri­aza­phosphaadamantane-κ*P*)ruthenium(II)] tetra­bromido­(ethanol/methanol-κ*O*)nitrosylrhenate(II), [Ru(CN)(C_5_H_5_)_2_(C_6_H_12_N_3_P)_4_][ReBr_4_(NO)(CH_4_O)_0.5_(C_2_H_6_O)_0.5_] or [RuCp(PTA)_2_–μ-CN–1κ*C*:2κ^2^ *N*-RuCp(PTA)_2_][Re(NO)Br_4_(EtOH)_0.5_(MeOH)_0.5_] (PTA = 3,5,7-tri­aza­phosphaadaman­tane) was obtained and characterized by single-crystal X-ray diffraction, elemental analysis and infrared spectroscopy. The title salt was obtained by liquid–liquid diffusion of methanol/DMSO solutions of (NBu_4_)[Re(NO)Br_4_(EtOH)] and [(PTA)_2_CpRu–μ-CN–1κ*C*:2κ^2^ *N*-RuCp(PTA)_2_](CF_3_SO_3_). The Ru^II^ and Re^II^ independent moieties correspond to a binuclear and mononuclear complex ion, respectively. A deep geometrical parameter analysis was performed, and no significant differences were found with earlier reports containing similar mol­ecules. The magnetic properties were investigated in the temperature range 2.0–300 K, and the complex behaves as a quasi-magnetically isolated spin doublet with weak anti­ferromagnetic inter­actions.

## Abstract 2

In this work, we present the complex salt [RuCp(PTA)_2_–μ-CN–1κ*C*:2κ^2^ *N*-RuCp(PTA)_2_][Re(NO)Br_4_(EtOH)_0.5_(MeOH)_0.5_]. The synthesis, single-crystal X-ray crystal structure, and magnetic properties are discussed.

## Chemical context   

Ruthenium-arene-PTA (PTA = 3,5,7-tri­aza-phosphaadamantane) or RAPTA complexes are known in inorganic medicinal chemistry for their potent anti­tumor activity *in vitro* and *in vivo*, constituting a potential alternative to platinum-based drugs (Antonarakis & Emadi, 2010 ▸; Gasser *et al.*, 2011 ▸; Liang *et al.*, 2017 ▸; Hey-Hawkins & Hissler, 2019 ▸). Furthermore, PTA presents variable denticity allowing it to act as a versatile building block towards the synthesis of coordination polymers with applications in other areas such as chemical catalysis (Darensbourg *et al.*, 1995 ▸; Scalambra *et al.*, 2017 ▸; Scalambra, Lopez-Sanchez *et al.*, 2020 ▸) and material science (Phillips *et al.*, 2004 ▸). Professor Romerosa’s group and coworkers have developed a family of water-soluble and air-stable organometallic polymers containing an ‘RuCp(PTA)_2_’ (Cp = Cyclo­penta­dien­yl) fragment. Most of them fit the general formula [{RuCp(PTA)_2_–μ-CN–1κ*C*:2κ^2^ *N*-RuCp(PTA)_2_}-μ-*MX_m_*]*_n_ (M* = Cd, Ag, Ni, Au, Co; *X* = halide or pseudohalide) (Serrano Ruiz *et al.*, 2008 ▸; Lidrissi *et al.*, 2005 ▸; Scalambra *et al.*, 2015 ▸, 2018 ▸; Scalambra, Sierra-Martin *et al.*, 2020 ▸). These polymers show exciting properties such as the formation of structured microparticles, amorphization under low pressures (Scalambra *et al.*, 2015 ▸, 2016 ▸), the formation of layered structures that can be exfoliated in ultra-thin 3D layers (Scalambra, Sierra-Martin *et al.*, 2020 ▸), the formation of gels in the presence of water (Sierra-Martin *et al.*, 2018 ▸, 2019 ▸; Serrano Ruiz *et al.*, 2008 ▸) or the capacity to capture water mol­ecules in nanochannels (Scalambra *et al.*, 2017 ▸). The described polymers include a wide variety of arrangements from one to three dimensions, and they may be classified as a new class of materials lying between metal–organic frameworks (MOFs) and infinite coordination polymers (ICPs) (Spokoyny *et al.*, 2009 ▸). The preparation mostly involves the use of the bimet­allic precursor RuCp(PTA)_2_–μ-CN–1κ*C*:2κ^2^ *N*-RuCp(PTA)_2_](CF_3_SO_3_) in the reaction with other transition-metal cation salts or complexes, in an easy, robust and reproducible method (Serrano-Ruiz *et al.*, 2014 ▸).

On top of that, rhenium nitrosyl complexes applications are widely recognized: catalysis, production of organo­nitro­gen compounds, pollutant control, nitric oxide release drugs, assembly of devices with novel optical and magnetic properties, among other uses (Machura, 2005 ▸; Jiang *et al.*, 2011 ▸; Probst *et al.*, 2009 ▸; Ghosh *et al.*, 2014 ▸; Dilworth, 2021 ▸). Kremer’s group has performed a thorough magnetic study of a series of complexes (NBu_4_)[Re^II^(NO)Br_4_(*L*)] (*L* is an *N*,*O* or *P*-donor neutral ligand) (Pacheco *et al.*, 2013 ▸; Pacheco, Cuevas, González-Platas, Lloret *et al.*, 2015 ▸). The low-spin outer 5*d* ^5^ shell results in strong spin-orbit inter­actions giving rise to a significant magnetic anisotropy, an essential feature for the potential construction of mol­ecule-based magnets (Wang *et al.*, 2011 ▸). In this work, we present the complex salt [RuCp(PTA)_2_–μ-CN–1κ*C*:2κ^2^ *N*-RuCp(PTA)_2_][Re(NO)Br_4_(EtOH)_0.5_(MeOH)_0.5_]. The synthesis, single crystal X-ray crystal structure, and magnetic properties are discussed.
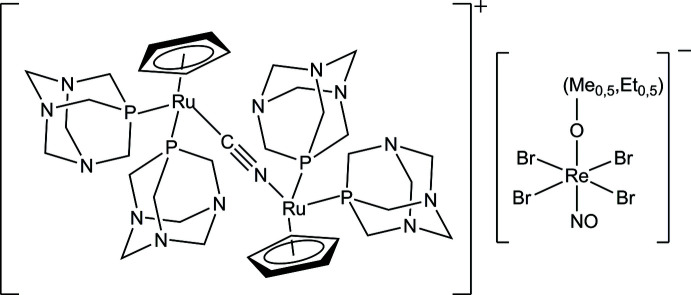


## Structural commentary   

**Figure 1:**
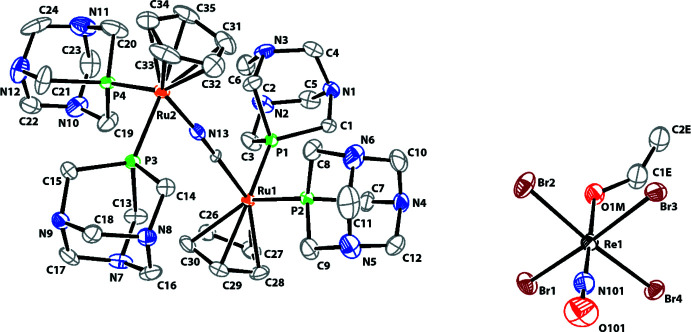
The asymmetric unit of the title compound, including atom labelling. Displacement ellipsoids are drawn at the 50% probability level. For clarity, H atoms have been omitted.

The mol­ecular structure of [RuCp(PTA)_2_–μ-CN–1κ*C*:2κ^2^ *N*-RuCp(PTA)_2_][Re(NO)Br_4_(EtOH)_0.5_(MeOH)_0.5_] consists of discrete [RuCp(PTA)_2_–μ-CN–1κ*C*:2κ^2^ *N*-RuCp(PTA)_2_]^+^ cations and [Re(NO)Br_4_(EtOH)_0.5_(MeOH)_0.5_]^−^ anions (Fig. 1 ▸), which coform the asymmetric unit.

The cation is an homobinuclear Ru^II^ complex with two piano-stool fashion {RuCp(PTA)_2_} moieties that are linked by a –CN– bridging ligand. The {CpRu(PTA)_2_}^+^ moieties in each Ru_2_ unit exhibit a transoid arrangement related to the Ru—C≡N—Ru axis. The Ru1—C25 and Ru2—N13 distances are 2.008 (7) and 2.030 (8) Å, respectively. The Ru—CN—Ru arrangement is practically linear: <(Ru1—C25—N13) = 175.5 (7)° and <(C25—N13—Ru2) = 176.3 (7)°. The C≡N bond length of the cyano group is 1.14 (1) Å. The distances from the centroid of each Cp ligand to the respective ruthenium atom are 1.886 Å (Cp—Ru1) and 1.878 (Cp—Ru2). The Ru—P_PTA_ distances are in the range 2.243 (2)–2.281 (2) Å, which is in agreement with those found in similar compounds.

The complex anion is constituted by an Re^II^ atom and displays a distorted octa­hedral geometry formed by four bromide ions in the equatorial plane, one nitro­gen atom from the nitrosyl ligand, and one oxygen atom from an –OH group in apical positions. The –OH group comes from a methanol or an ethanol mol­ecule, both with an s.o.f. of 0.5. The O1M and C1E atomic positions are the same for both the MeOH and the EtOH ligand. The Re1—O1m—C1e angle is 128.3 (6)°. The NO group is practically linear with an O101—N101—Re1 angle of 178.6 (10)°. The three atoms are also aligned with the O1M atom of the alcohol ligand, exhibiting a N101—Re1—O1M angle of 178.9 (3)°. The rhenium atom is shifted from the main plane of Br ligands towards the apical NO group by 0.157 Å.

## Supra­molecular features   

**Figure 2:**
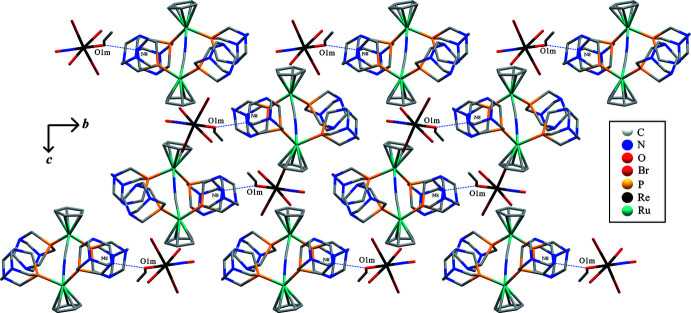
View along the *a* axis of the title compound, with the O1M—H⋯N8 contacts (see Table 1 ▸ for details) represented by blue dashed lines. For clarity, H atoms have been omitted.

The complex crystallizes in the monoclinic *P*2_1_/c space group. The cations inter­connect adjacent anions *via* O—H⋯N hydrogen bonds and C—H⋯Br inter­actions, forming an infinite three-dimensional framework (Table 1 ▸). The O—H⋯N inter­actions are given along the *bc* plane and are defined by O1m as the donor atom from the MeOH/EtOH ligand and N8^i^ atom from a PTA ligand at (*x* − 1, *y*, *z*) (Fig. 2 ▸). The H1M⋯N8^i^ and O1M⋯N8^i^ distances are 1.88 and 2.709 (9) Å, respectively. The angle defined by O1M—H1M⋯N8^i^ is 165.5°.

The remaining hydrogen bonds are found between the PTA ligands from one cationic unit [RuCp(PTA)_2_–μ-CN–1κ*C*:2κ^2^ *N*-RuCp(PTA)_2_]^+^ and bromides from [Re(NO)Br_4_(EtOH)_0.5_(MeOH)_0.5_]^−^ units. The multiplicity and lack of defined directionality in the hydrogen-bond network are related to the fact that the major forces that stabilize the crystal are of electrostatic origin. The C—H⋯Br and the C⋯Br distances range from 2.53–3.12 Å and 3.208 (11)–3.944 (12) Å, respectively. The hydrogen-bond angle involving the C—H⋯Br atoms vary between 127 and 169°. These geometrical values are in concordance with weak hydrogen-bonding inter­actions (Desiraju, 1995 ▸; Metrangolo *et al.*, 2006 ▸; Steed & Atwood, 2009 ▸). The effect of the combined weak C—H⋯Br bonds and their effect on the crystal assembly can be as significant as that of the strong inter­actions (Desiraju & Steiner, 2001 ▸). The C2E—H⋯N6 bond is probably negligible because of the low energy expected for all C—H bonds (Steed & Atwood, 2009 ▸) and particularly considering the C2E 50% atomic site occupation.

## Hirshfeld analysis   

**Figure 3:**
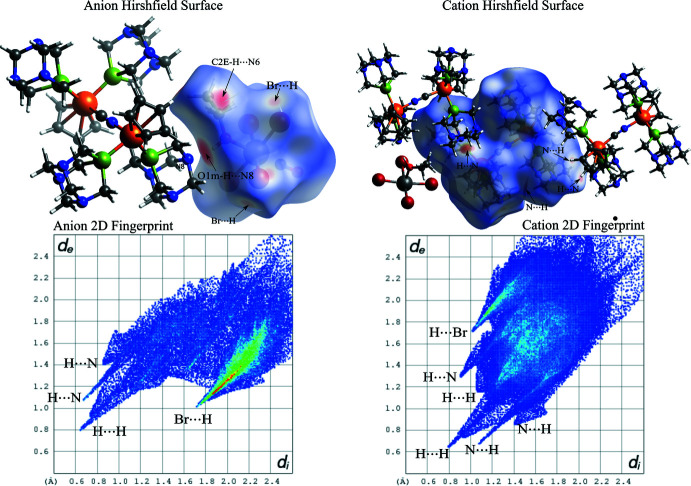
Projections of *d*_norm_ mapped on Hirshfeld surfaces, showing the inter­actions between mol­ecules and the two-dimensional (*d*_i_,*d*_e_) fingerprint plot for the anionic unit [Re(NO)Br_4_(EtOH)_0.5_(MeOH)_0.5_]^−^ (left) and the cationic unit [RuCp(PTA)_2_–μ-CN–1κ*C*:2κ^2^ *N*-RuCp(PTA)_2_]^+^ (right).

To further understand the inter­molecular inter­actions between the ionic complexes within the crystal structure, a Hirshfeld surface (Spackman & Jayatilaka, 2009 ▸) was constructed around each ion. In addition, a 2D fingerprint plot analysis (Spackman & McKinnon, 2002 ▸) was performed for each case. *Crystal Explorer17* (Turner *et al.*, 2017 ▸) was used to determine the surface and construct the plots. The Hirshfeld surfaces of both the anion and cation are illustrated in Fig. 3 ▸ (left) and 3 (right), respectively, showing surfaces that have been mapped over a *d*_norm_ range of −0.6854 to 1.6426 a.u. (McKinnon *et al.*, 2007 ▸). The color code employed for *d*_norm_ is red for the shortest *d*_norm_ and blue for the longest *d*_norm_. Red spots in the surface correspond to the shortest contacts within the surface, indicating the formation of inter­molecular bonds as those detailed in the previous section (supra­molecular features).

**Figure 4:**
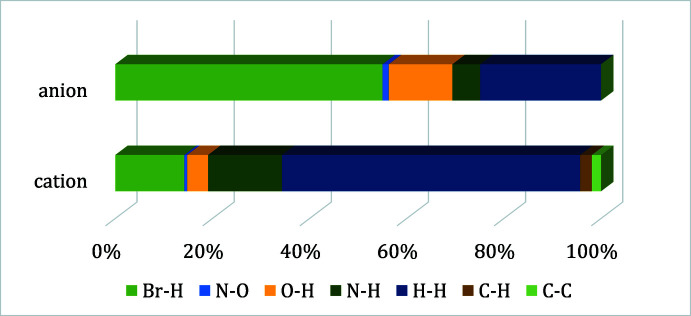
Relative contributions to Hirshfeld surface area for the close mol­ecular contacts.

The anion Hirshfeld surface shows how the most significant inter­action is due to the O1m—H⋯N8 bond, which is illustrated by bright-red spots in Fig. 3 ▸ (left), while the weaker spot corresponds to the C2E—H⋯N6 bond. What is more, the other minor red spots can be identified as Br⋯H inter­actions. These red spots (and thus the inter­ionic inter­actions) can be correlated with the spikes observed in the two-dimensional fingerprint plots. In fact, the anion fingerprint for all inter­actions exhibits characteristic spikes in the region 1.8 Å < *d*_i_ + *d*_e_ < 2.8 Å resulting from H⋯N and Br⋯H inter­actions. There is a high-density area close to the Br⋯H spike, indicating a significant number of Br⋯H contacts in the crystal structure. In addition, the broad central spike extending up to the (*d*_i_,*d*_e_) region of (0.65 Å, 0.78 Å) reflects the significant amount of H⋯H contacts in the structure. Nevertheless, it is important to point out that the H⋯H contacts are usually difficult to localize in the Hirshfeld surface as they are spread all over the crystal packing. The Hirshfeld surface analysis for the cationic unit and its fingerprint also shows how H⋯N, N⋯H, H⋯Br, and H⋯H contacts surround the [RuCp(PTA)_2_–μ-CN–1κ*C*:2κ^2^ *N*-RuCp(PTA)_2_] unit. The relative contributions of the different inter­molecular contacts to the Hirshfeld area for both ions are shown in Fig. 4 ▸. In the anion, the major contributors (∼93%) are from Br⋯H, O⋯H and H⋯H contacts while in the cation, the Hirshfeld area is accounted mostly by the Br⋯H, N⋯H and H⋯H contacts (over 90%).

## Database survey   

A search in the Cambridge Structural Database (CSD) version 5.42 in the last update of February 2021 (Groom *et al.*, 2016 ▸) for similar structures containing the anion and cation was performed. The {(PTA)_2_CpRu-μ-CN-RuCp(PTA)_2_} moiety has been reported previously, once as an independent cationic unit in VOHCUS (Serrano-Ruiz *et al.*, 2014 ▸) as well as a fragment within polynuclear polymeric structures CEQPEW (Scalambra *et al.*, 2018 ▸), EDONET (Scalambra *et al.*, 2016 ▸), GUVZUV (Scalambra, Sierra-Martin *et al.*, 2020 ▸) and XADHES (Scalambra *et al.*, 2015 ▸).

Regarding the anionic unit, examples of crystal structures containing tetra­bromo­nitro­sylrhenium(II) complexes are scarce. The CSD search yielded 19 hits. In all of them, the rhenium coordination sphere exhibits an octa­hedral geometry, with a practically lineal {Re—NO} unit and a π-acceptor ligand such as phosphine or aromatic amines, usually coordinating *trans*- to the –NO group. The found π-acceptor ligands include: MeCN (Ciani *et al.*, 1975 ▸), EtOH (Ciani *et al.*, 1975 ▸), pyrazine (Pacheco *et al.*, 2013 ▸, 2014 ▸; Pacheco, Cuevas, González-Platas, & Kremer, 2015 ▸), nitrosyl (Mronga *et al.*, 1982 ▸), tri­cyclo­hexyl­phosphine and triiso­propyl­phosphine (Jiang *et al.*, 2010 ▸), nicotinic acid and nicotinate anion (Pacheco, Cuevas, González-Platas, Lloret *et al.*, 2015 ▸), pyridine, pyrimidine and pyridazine (Pacheco *et al.*, 2013 ▸). All Re—Br distances observed in the complex reported herein, as well as the Re—N and N—O distances found, agree with those found for previously reported structures (see Figs. 1–3 in the supporting information).

A search in the CSD for complexes containing a metal ion coordinating a MeOH mol­ecule yielded 13705 structures with the *M*—O—C angle lying in the range 123.333–130.865° (without considering possible outlier values). The same angle for metals coordinating an EtOH is in the range 124.464–132.412° (without considering possible outliers), in a total of 3503 reported structures. There are only five structures reported in the database containing ethanol coordinating to a rhenium atom, ABENRE (Ciani *et al.*, 1975 ▸), PIXTOF (Masood & Hodgson, 1994 ▸), GEMVUR (Ikeda *et al.*, 2012 ▸), EGAVEP (Hołyńska & Lis, 2014 ▸) and PIMRAH (Pino-Cuevas *et al.*, 2018 ▸). In those, the Re—O—C angles vary between 115.8 (4) and 135 (1)°. The same search but for Re-OHMe complexes yielded 15 structures, with the Re—O—C angles in the 121.232–133.389° range. The only reported crystal structure in the CSD containing the [Re(NO)Br_4_(EtOH)]^−^ unit dates back to 1975 (ABENRE; Ciani *et al.*, 1975 ▸). On the other hand, this is the first report of a crystal structure evidencing the coordination of a methanol mol­ecule substituting ethanol.

Given that C—H⋯Br bonds account for a significant fraction of inter­molecular contacts, as seen in section 4, a search was conducted involving this bonding scheme to check if the values presented in this article are within the bin frequently encountered in transition-metal compounds. The search restrained metal–Br⋯H distances to be lower than the sum of the vdW radius (∼3.5 Å). Compounds containing a C—Br⋯H angle of less than 90° were discarded, as the hydrogen atom in the hydrogen bond must not point away from the acceptor atom (Aakeröy *et al.*, 1999 ▸). The search resulted in 36099 hits from 12143 structures. The histograms of C⋯Br distances and C—H⋯Br angles (Figs. 4 and 5 in the supporting information) confirm that these H⋯Br contacts, considering the distance/angle criteria, can be identified as hydrogen bonds (Aakeröy *et al.*, 1999 ▸; Metrangolo *et al.*, 2006 ▸; Shimpi *et al.*, 2007 ▸; Zhang *et al.*, 2008 ▸).

## Magnetic measurements   

Magnetic susceptibility measurements on polycrystalline samples were carried out with a Superconducting Quantum Inter­ference Design (SQUID) magnetometer in the temperature range 2.0–300 K. In order to avoid saturation phenomena, we used external *dc* magnetic fields of 500 G (*T* < 20 K) and 5000 G (*T* ≥ 50 K). Experimental susceptibilities were carefully corrected for the diamagnetism of the holder (gelatine capsule) and constituent atoms by applying Pascal’s constants.

**Figure 5:**
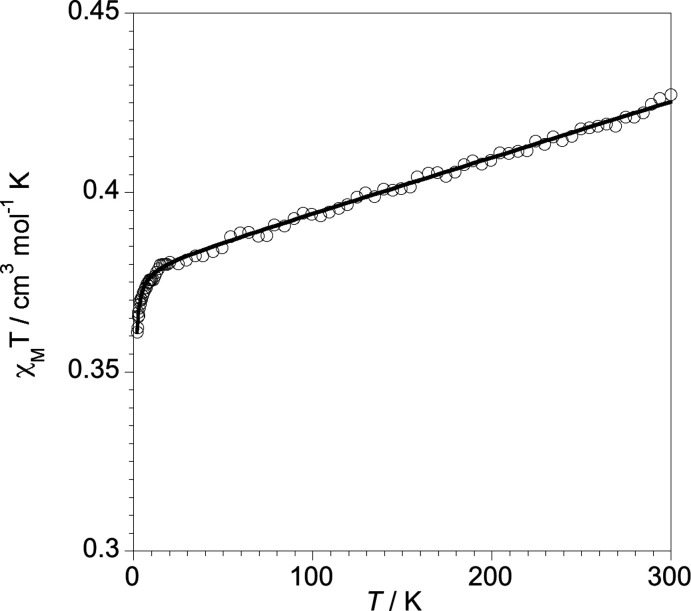
χ*_M_T versus T* plot for the title compound.

The magnetic behaviour of [RuCp(PTA)_2_–μ-CN–1κ*C*:2κ^2^ *N*-RuCp(PTA)_2_][Re(NO)Br_4_(EtOH)_0.5_(MeOH)_0.5_] is shown in Fig. 5 ▸ in the form of a χ*_M_T versus T* plot where χ_*M*_ is the molar magnetic susceptibility per one Re^II^ ion and *T* the absolute temperature. As expected, a straight line is observed for this compound (Pacheco *et al.*, 2013 ▸). The thermal dependence of χ*_M_T* is in line with one unpaired electron (*S* = ½) and a temperature independent paramagnetic contribution (TIP). The χ*_M_T* value at room temperature is higher than that expected for an *S* = ½ with *g* = 2.0 (0.375 cm^3^ K mol^−1^) due to the temperature-independent paramagnetism (*TIP*). The slight decrease below 10 K must be attributed to very weak inter­molecular anti­ferromagnetic (AF) inter­actions between the [Re(NO)Br_4_(EtOH)_0.5_(MeOH)_0.5_]^−^ anions.

In this sense, we use equation (1), with *S* = ½, to fit the experimental data.


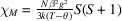
 (1)

Best-fit parameters were *g* = 2.01 (1), *TIP* = 155 (3) 10^−6^ cm^3^ mol^−1^ and *θ* = – 0.100 (1) K. The calculated *g* and *TIP* values are very close to those observed for similar complexes previously reported (Pacheco *et al.*, 2013 ▸; Pacheco, Cuevas, González-Platas, Lloret *et al.*, 2015 ▸). However, the Weiss parameter (inter­molecular anti­ferromagnetic inter­action), *θ*, is lower, indicating that the paramagnetic anion is much more isolated, probably due to the vast diamagnetic counter-ion.

## Synthesis and crystallization   

### Experimental details   

(NBu_4_)[Re(NO)Br_4_(EtOH)] and [RuCp(PTA)_2_–μ-CN–1κ*C*:2κ^2^ *N*-RuCp(PTA)_2_](CF_3_SO_3_) were prepared as previously reported (Pacheco *et al.*, 2013 ▸; Serrano-Ruiz *et al.*, 2014 ▸). Solvents employed in the synthesis were purchased from commercial sources and used without further purification. Elemental analyses (C, H, N, S) were performed using a Flash 2000 (Thermo Scientific) elemental analyser. The IR spectra were recorded as 1% KBr pellets on FTIR Shimadzu Prestige-21 spectrophotometer in the range 4000-400 cm^−1^.

### Synthesis   

A solution of (NBu_4_)[Re(NO)Br_4_(EtOH)] (0.012 mmol, 10 mg) dissolved in 5 mL of a methanol–DMSO (400:1, *v*/*v*) mixture was layered in an test tube with a solution of [RuCp(PTA)_2_–μ-CN–1κ*C*:2κ^2^ *N*-RuCp(PTA)_2_](CF_3_SO_3_) (0.012 mmol, 13 mg) in 5 mL of the same methanol–DMSO mixture; *ca* 5 mL of the solvent mixture should be added between the two reactant layers to decrease diffusion time. Deep reddish-brown plate-like crystals, suitable for single crystal X-ray diffraction were obtained after one week. The product was filtered and washed by deca­ntation with methanol. Yield: 24%. Analysis calculated for Ru_2_C_36.5_N_14_Re_1_O_2_Br_4_H_63_P_4_: C, 28.07; H, 4.07; N, 12.56; S. 0,00%. Found: C, 27.18; H, 4.39; N, 12.53; S. 0,00%. Selected IR absorption bands (KBr, ν_max_/cm^−1^): 3413[*s*, *br*, ν_s_(–OH)], 2922(*w*), 2114[*m*, ν_s_(*μ*–N≡C)], 1759[*s*, ν_s_ (–NO)], 1280(*m*), 1242(*s*), 1097(*m*), 1016(*s*), 970(*s*), 948(*s*), 833(*w*), 744(*w*), 574(*m*), 480(*m*).

## Refinement   

Crystal data, data collection and structure refinement details are summarized in Table 2 ▸. The C-bound H atoms were included in calculated positions and treated as riding: C—H distance between 0.94 and 0.98 Å with *U*_iso_(H) = 1.2*U*_eq_(C). Methanol/ethanol coordinating mol­ecules were treated as positionally disordered utilizing the PART instruction with occupancy fixed to 0.5 applied to C1E, C1M, and C2E. C1M and C1E were constrained to occupy equivalent positions. Meanwhile, C2E was located in the Fourier difference map and refined freely.

## Supplementary Material

Crystal structure: contains datablock(s) I. DOI: 10.1107/S2056989021006381/ex2046sup1.cif

Structure factors: contains datablock(s) I. DOI: 10.1107/S2056989021006381/ex2046Isup2.hkl

Histogram showing the number of Re-Br distances hexacoordinated nitrosylrhenium complexes found in the database survey (section 5). DOI: 10.1107/S2056989021006381/ex2046sup3.png

Histogram showing the number of Re-N(NO) distances in hexacoordinated nitrosylrhenium complexes found in the database survey (section 5). DOI: 10.1107/S2056989021006381/ex2046sup4.png

Histogram showing the number of N-O nitrosyl distances in hexacoordinated nitrosylrhenium complexes found in the database survey (section 5). DOI: 10.1107/S2056989021006381/ex2046sup5.png

Histogram showing the number of C...Br distances in hexacoordinated nitrosylrhenium complexes found in the database survey (section 5). DOI: 10.1107/S2056989021006381/ex2046sup6.png

Histogram showing the number of C...H-Br angles in hexacoordinated nitrosylrhenium complexes found in the database survey (section 5). DOI: 10.1107/S2056989021006381/ex2046sup7.png

CCDC reference: 2075886

Additional supporting information:  crystallographic information; 3D view; checkCIF report

## Figures and Tables

**Table 1:** Hydrogen-bond geometry (Å, °)

*D*—H⋯*A*	*D*—H	H⋯*A*	*D*⋯*A*	*D*—H⋯*A*
C10—H10*A*⋯Br3^i^	0.97	3.12	3.944 (12)	143
C10—H10*B*⋯Br2	0.97	2.83	3.709 (10)	150
C1—H1*B*⋯Br4^ii^	0.97	3.03	3.967 (9)	163
C7—H7*B*⋯N9^iii^	0.97	2.59	3.309 (11)	131
C8—H8*A*⋯Br3^i^	0.97	2.89	3.772 (12)	151
C4—H4*B*⋯Br3^i^	0.97	3.10	4.062 (10)	169
C5—H5*A*⋯Br1^ii^	0.97	3.10	3.918 (10)	143
C18—H18*A*⋯N4^iv^	0.97	2.53	3.208 (11)	127
C18—H18*B*⋯Br2^v^	0.97	2.92	3.858 (9)	163
C19—H19*B*⋯Br1^vi^	0.97	3.09	3.938 (11)	147
C22—H22*B*⋯Br1^vi^	0.97	3.00	3.861 (10)	148
C23—H23*A*⋯Br4^vii^	0.97	3.10	4.007 (12)	156
C24—H24*A*⋯Br3^vii^	0.97	2.98	3.799 (11)	143
O1M—H1M⋯N8^iii^	0.85	1.88	2.709 (9)	166
C1E*B*—H101⋯Br3	0.97	2.80	3.527 (13)	132
C2E—H2E3⋯N6^i^	0.96	2.36	3.15 (3)	140

Symmetry codes: (i) -x, -y, -z; (ii) -x, y+{\script{1\over 2}}, -z+{\script{1\over 2}}; (iii) x-1, y, z; (iv) x+1, y, z; (v) -x+1, -y, -z; (vi) -x+1, y+{\script{1\over 2}}, -z+{\script{1\over 2}}; (vii) x+1, y+1, z.

**Table 2:** Experimental details

Crystal data	
Chemical formula	[Ru(CN)(C_5_H_5_)_2_(C_6_H_12_N_3_P)_4_]_2_[ReBr_4_(NO)(CH_4_O)_0.5_(C_2_H_6_O)_0.5_]_2_
*M*_r_	3123.73
Crystal system, space group	Monoclinic, *P*2_1_/*c*
Temperature (K)	296
*a*, *b*, *c* (Å)	12.6027 (4), 17.7075 (6), 23.0252 (9)
β (°)	101.914 (1)
*V* (Å^3^)	5027.7 (3)
*Z*	2
Radiation type	Mo *K*α
μ (mm^−1^)	6.35
Crystal size (mm)	0.48 × 0.10 × 0.03

Data collection	
Diffractometer	Bruker D8 venture diffractometer
Absorption correction	Multi-scan (*SADABS*; Krause *et al.*, 2015 ▸)
*T*_min_, *T*_max_	0.485, 0.751
No. of measured, independent and observed [*I* > 2σ(*I*)] reflections	56882, 8565, 6494
*R*_int_	0.079
(sin θ/λ)_max_ (Å^−1^)	0.589

Refinement	
*R*[*F*^2^ > 2σ(*F* ^2^)], *wR*(*F* ^2^), *S*	0.044, 0.118, 0.99
No. of reflections	8565
No. of parameters	572
H-atom treatment	H-atom parameters constrained
Δρ_max_, Δρ_min_ (e Å^−3^)	1.45, −1.34

Computer programs: *APEX2* (Bruker, 2007 ▸), *SAINT* (Bruker, 2013 ▸), *SHELXT2014/4* (Sheldrick, 2015*a* ▸), *SHELXL2018/3* (Sheldrick, 2015*b* ▸), *ORTEP-3 for Windows* (Farrugia, 2012 ▸) and *Mercury* (Macrae *et al.*, 2020 ▸).

## supplementary crystallographic information

### Crystal data

**Table:**

[Ru(CN)(C_5_H_5_)_2_(C_6_H_12_N_3_P)_4_]_2_[ReBr_4_(NO)(CH_4_O)_0.5_(C_2_H_6_O)_0.5_]_2_	*F*(000) = 3036
*M**_r_* = 3123.73	*D*_x_ = 2.063 Mg m^−^^3^
Monoclinic, *P*2_1_/*c*	Mo *K*α radiation, λ = 0.71073 Å
*a* = 12.6027 (4) Å	Cell parameters from 125 reflections
*b* = 17.7075 (6) Å	θ = 3.1–16.9°
*c* = 23.0252 (9) Å	µ = 6.35 mm^−^^1^
β = 101.914 (1)°	*T* = 296 K
*V* = 5027.7 (3) Å^3^	Prism, orange
*Z* = 2	0.48 × 0.10 × 0.03 mm

### Data collection

**Table:**

Bruker D8 venture diffractometer	8565 independent reflections
Radiation source: sealed tube, SIEMENS KFFMO2K-90C model 10190380	6494 reflections with *I* > 2σ(*I*)
Curved graphite monochromator	*R*_int_ = 0.079
Detector resolution: 10.4167 pixels mm^-1^	θ_max_ = 24.7°, θ_min_ = 2.8°
φ and ω scans	*h* = −14→14
Absorption correction: multi-scan (SADABS; Krause *et al.*, 2015)	*k* = −20→20
*T*_min_ = 0.485, *T*_max_ = 0.751	*l* = −27→27
56882 measured reflections	

### Refinement

**Table:**

Refinement on *F*^2^	Primary atom site location: iterative
Least-squares matrix: full	Secondary atom site location: difference Fourier map
*R*[*F*^2^ > 2σ(*F*^2^)] = 0.044	Hydrogen site location: mixed
*wR*(*F*^2^) = 0.118	H-atom parameters constrained
*S* = 0.99	*w* = 1/[σ^2^(*F*_o_^2^) + (0.0568*P*)^2^ + 32.4246*P*] where *P* = (*F*_o_^2^ + 2*F*_c_^2^)/3
8565 reflections	(Δ/σ)_max_ < 0.001
572 parameters	Δρ_max_ = 1.45 e Å^−^^3^
0 restraints	Δρ_min_ = −1.34 e Å^−^^3^

### Special details

**Table:**

Geometry. All esds (except the esd in the dihedral angle between two l.s. planes) are estimated using the full covariance matrix. The cell esds are taken into account individually in the estimation of esds in distances, angles and torsion angles; correlations between esds in cell parameters are only used when they are defined by crystal symmetry. An approximate (isotropic) treatment of cell esds is used for estimating esds involving l.s. planes.

### Fractional atomic coordinates and isotropic or equivalent isotropic displacement parameters (Å^2^)

**Table:**

	*x*	*y*	*z*	*U*_iso_*/*U*_eq_	Occ. (<1)
Re1	−0.05570 (3)	−0.18588 (2)	0.11322 (2)	0.03581 (11)	
Ru1	0.48233 (5)	0.25106 (4)	0.22334 (3)	0.02682 (15)	
Ru2	0.66214 (5)	0.27893 (4)	0.04193 (3)	0.02792 (16)	
Br1	0.04222 (9)	−0.12082 (7)	0.20632 (4)	0.0584 (3)	
Br2	0.04855 (10)	−0.10735 (7)	0.05355 (5)	0.0667 (3)	
Br3	−0.16673 (10)	−0.23895 (6)	0.01809 (4)	0.0595 (3)	
Br4	−0.17972 (8)	−0.24939 (6)	0.17119 (4)	0.0518 (3)	
P1	0.35033 (15)	0.33600 (12)	0.18506 (8)	0.0268 (4)	
P2	0.40014 (16)	0.15060 (12)	0.17422 (9)	0.0318 (5)	
P3	0.79305 (16)	0.20690 (12)	0.09819 (8)	0.0279 (4)	
P4	0.72619 (16)	0.39303 (12)	0.07922 (9)	0.0294 (4)	
O101	0.0961 (8)	−0.3111 (6)	0.1265 (5)	0.110 (4)	
N1	0.1390 (6)	0.3853 (4)	0.1485 (3)	0.0434 (18)	
N2	0.2668 (7)	0.4759 (5)	0.2046 (4)	0.054 (2)	
N3	0.2747 (6)	0.4471 (5)	0.1013 (4)	0.050 (2)	
N4	0.2311 (6)	0.0505 (4)	0.1466 (4)	0.055 (2)	
N6	0.3384 (9)	0.0693 (5)	0.0709 (4)	0.072 (3)	
N7	0.8856 (6)	0.1427 (4)	0.2069 (3)	0.048 (2)	
N8	0.8650 (5)	0.0578 (4)	0.1213 (3)	0.0384 (17)	
N9	1.0030 (5)	0.1578 (4)	0.1354 (3)	0.0389 (17)	
N10	0.7525 (8)	0.5001 (5)	0.1692 (4)	0.063 (2)	
N11	0.6862 (10)	0.5468 (5)	0.0695 (5)	0.079 (3)	
N12	0.8770 (8)	0.5093 (5)	0.1015 (5)	0.068 (3)	
C10	0.2299 (9)	0.0593 (6)	0.0845 (6)	0.075 (4)	
H10A	0.185518	0.102699	0.069690	0.090*	
H10B	0.196148	0.015088	0.063481	0.090*	
C11	0.4070 (11)	0.0061 (7)	0.0987 (7)	0.088 (4)	
H11A	0.379585	−0.040178	0.078516	0.106*	
H11B	0.479829	0.013789	0.092099	0.106*	
N13	0.5859 (5)	0.2744 (4)	0.1114 (3)	0.0363 (16)	
N101	0.0350 (7)	−0.2596 (5)	0.1217 (4)	0.057 (2)	
C1	0.2033 (6)	0.3182 (5)	0.1677 (4)	0.043 (2)	
H1A	0.187494	0.280371	0.136628	0.052*	
H1B	0.181946	0.297768	0.202646	0.052*	
C2	0.3562 (7)	0.3865 (5)	0.1154 (4)	0.044 (2)	
H2A	0.427904	0.408071	0.118761	0.053*	
H2B	0.344982	0.350473	0.082962	0.053*	
C3	0.3459 (8)	0.4201 (5)	0.2319 (4)	0.053 (3)	
H3A	0.329067	0.404229	0.269273	0.063*	
H3B	0.417142	0.443326	0.240483	0.063*	
C7	0.2643 (7)	0.1199 (6)	0.1800 (5)	0.053 (3)	
H7A	0.262715	0.111570	0.221448	0.064*	
H7B	0.212869	0.159678	0.165259	0.064*	
C8	0.3864 (9)	0.1426 (6)	0.0934 (4)	0.057 (3)	
H8A	0.340877	0.183277	0.074070	0.069*	
H8B	0.457243	0.147751	0.083554	0.069*	
C9	0.4687 (8)	0.0597 (5)	0.1959 (6)	0.064 (3)	
H9A	0.542754	0.062672	0.190220	0.077*	
H9B	0.471397	0.050991	0.237786	0.077*	
N5	0.4139 (8)	−0.0044 (5)	0.1616 (5)	0.071 (3)	
C4	0.1629 (7)	0.4158 (6)	0.0938 (4)	0.048 (2)	
H4A	0.111021	0.455341	0.079097	0.057*	
H4B	0.153739	0.376066	0.064165	0.057*	
C5	0.1567 (8)	0.4440 (6)	0.1932 (4)	0.055 (3)	
H5A	0.142735	0.423527	0.229964	0.066*	
H5B	0.105007	0.484405	0.180683	0.066*	
C6	0.2891 (9)	0.5031 (5)	0.1497 (5)	0.061 (3)	
H6A	0.363300	0.521225	0.156964	0.074*	
H6B	0.242057	0.545789	0.136630	0.074*	
C12	0.3026 (9)	−0.0125 (5)	0.1703 (5)	0.064 (3)	
H12A	0.304399	−0.016960	0.212516	0.077*	
H12B	0.272252	−0.058858	0.151518	0.077*	
C13	0.8036 (8)	0.1997 (5)	0.1790 (4)	0.043 (2)	
H13A	0.823328	0.248627	0.196916	0.051*	
H13B	0.733433	0.186003	0.186840	0.051*	
C14	0.7814 (7)	0.1043 (5)	0.0826 (4)	0.038 (2)	
H14A	0.710386	0.087306	0.087148	0.046*	
H14B	0.786260	0.095862	0.041597	0.046*	
C15	0.9390 (6)	0.2153 (5)	0.0987 (4)	0.0356 (19)	
H15A	0.950021	0.210954	0.058339	0.043*	
H15B	0.964059	0.264882	0.113423	0.043*	
C16	0.8588 (8)	0.0680 (5)	0.1841 (4)	0.049 (2)	
H16A	0.785748	0.056039	0.188513	0.058*	
H16B	0.907554	0.032285	0.207982	0.058*	
C17	0.9918 (7)	0.1643 (6)	0.1968 (4)	0.049 (2)	
H17A	1.006169	0.216183	0.209553	0.059*	
H17B	1.046353	0.132840	0.221310	0.059*	
C18	0.9748 (7)	0.0812 (5)	0.1149 (4)	0.041 (2)	
H18A	1.027553	0.046580	0.137183	0.049*	
H18B	0.979171	0.077487	0.073475	0.049*	
C19	0.7220 (10)	0.4204 (6)	0.1561 (4)	0.061 (3)	
H19A	0.649346	0.412212	0.162738	0.073*	
H19B	0.771012	0.388218	0.183392	0.073*	
C20	0.6489 (11)	0.4732 (6)	0.0421 (5)	0.077 (4)	
H20A	0.655078	0.473891	0.000761	0.092*	
H20B	0.572964	0.466475	0.043119	0.092*	
C21	0.8624 (9)	0.4296 (6)	0.0794 (6)	0.069 (3)	
H21A	0.915554	0.397600	0.104395	0.083*	
H21B	0.875592	0.427327	0.039412	0.083*	
C22	0.8613 (9)	0.5136 (6)	0.1613 (5)	0.065 (3)	
H22A	0.883435	0.563405	0.176712	0.078*	
H22B	0.909056	0.477218	0.185063	0.078*	
C23	0.6783 (10)	0.5495 (7)	0.1312 (6)	0.075 (4)	
H23A	0.691530	0.600858	0.145481	0.090*	
H23B	0.604921	0.536233	0.134178	0.090*	
C24	0.7978 (14)	0.5580 (7)	0.0637 (5)	0.087 (5)	
H24A	0.817520	0.610183	0.073033	0.104*	
H24B	0.802066	0.549376	0.022668	0.104*	
C25	0.5470 (5)	0.2690 (4)	0.1518 (3)	0.0225 (15)	
C26	0.5553 (8)	0.3156 (5)	0.3068 (4)	0.046 (2)	
H26	0.555150	0.370530	0.312146	0.055*	
C27	0.4748 (8)	0.2631 (7)	0.3181 (4)	0.053 (3)	
H27	0.409889	0.276314	0.333130	0.063*	
C28	0.5087 (9)	0.1890 (6)	0.3085 (4)	0.054 (3)	
H28	0.473025	0.142054	0.316367	0.065*	
C29	0.6103 (7)	0.1955 (6)	0.2917 (4)	0.045 (2)	
H29	0.656417	0.153412	0.284591	0.053*	
C30	0.6369 (7)	0.2727 (6)	0.2907 (4)	0.050 (3)	
H30	0.704052	0.292920	0.281675	0.060*	
C31	0.5113 (8)	0.2551 (7)	−0.0273 (4)	0.061 (3)	
H31	0.438182	0.249096	−0.019477	0.074*	
C32	0.5831 (10)	0.1982 (7)	−0.0281 (4)	0.062 (3)	
H32	0.568991	0.144879	−0.021077	0.075*	
C33	0.6779 (10)	0.2255 (8)	−0.0420 (4)	0.068 (4)	
H33	0.739328	0.195217	−0.048791	0.081*	
C34	0.6636 (10)	0.3038 (8)	−0.0516 (4)	0.074 (4)	
H34	0.713939	0.337921	−0.065857	0.089*	
C35	0.5567 (9)	0.3237 (7)	−0.0416 (4)	0.064 (3)	
H35	0.521157	0.373131	−0.047466	0.077*	
O1M	−0.1667 (5)	−0.0925 (4)	0.1036 (3)	0.0546 (17)	
H1m	−0.145698	−0.046869	0.108187	0.082*	
C1EB	−0.2859 (10)	−0.0942 (7)	0.0884 (6)	0.076 (3)	0.5
H101	−0.296961	−0.144748	0.071928	0.091*	0.5
H102	−0.301292	−0.099033	0.127783	0.091*	0.5
C2E	−0.3839 (19)	−0.0593 (14)	0.0584 (12)	0.076 (3)	0.5
H2e1	−0.405709	−0.021893	0.083727	0.114*	0.5
H2e2	−0.439669	−0.096818	0.048511	0.114*	0.5
H2e3	−0.372188	−0.035670	0.022699	0.114*	0.5
C1EA	−0.2859 (10)	−0.0942 (7)	0.0884 (6)	0.076 (3)	0.5
H1eA	−0.319631	−0.056188	0.058329	0.114*	0.5
H1eB	−0.320451	−0.093614	0.121497	0.114*	0.5
H1eC	−0.314444	−0.146063	0.065667	0.114*	0.5

### Atomic displacement parameters (Å^2^)

**Table:**

	*U*^11^	*U*^22^	*U*^33^	*U*^12^	*U*^13^	*U*^23^
Re1	0.0446 (2)	0.0355 (2)	0.02719 (18)	−0.00580 (16)	0.00707 (14)	−0.00453 (14)
Ru1	0.0259 (3)	0.0327 (3)	0.0212 (3)	−0.0019 (3)	0.0033 (2)	0.0023 (3)
Ru2	0.0300 (3)	0.0349 (4)	0.0180 (3)	0.0006 (3)	0.0031 (2)	−0.0007 (3)
Br1	0.0624 (6)	0.0696 (7)	0.0382 (5)	−0.0096 (5)	−0.0010 (4)	−0.0183 (5)
Br2	0.0934 (8)	0.0612 (7)	0.0558 (6)	−0.0312 (6)	0.0394 (6)	−0.0125 (5)
Br3	0.0914 (8)	0.0437 (6)	0.0345 (5)	−0.0140 (5)	−0.0078 (5)	−0.0047 (4)
Br4	0.0577 (6)	0.0529 (6)	0.0480 (6)	−0.0077 (5)	0.0184 (4)	0.0064 (4)
P1	0.0280 (10)	0.0299 (11)	0.0227 (10)	−0.0032 (9)	0.0056 (8)	−0.0018 (8)
P2	0.0303 (10)	0.0297 (11)	0.0330 (11)	−0.0023 (9)	0.0010 (8)	0.0038 (9)
P3	0.0303 (10)	0.0310 (11)	0.0222 (10)	0.0000 (9)	0.0054 (8)	0.0006 (8)
P4	0.0339 (11)	0.0292 (11)	0.0242 (10)	0.0016 (9)	0.0038 (8)	0.0049 (8)
O101	0.093 (7)	0.097 (8)	0.141 (10)	0.045 (6)	0.028 (6)	0.009 (7)
N1	0.037 (4)	0.046 (5)	0.044 (4)	0.003 (4)	0.002 (3)	0.003 (4)
N2	0.063 (5)	0.046 (5)	0.045 (5)	0.015 (4)	−0.005 (4)	−0.015 (4)
N3	0.056 (5)	0.047 (5)	0.051 (5)	0.014 (4)	0.018 (4)	0.022 (4)
N4	0.043 (4)	0.033 (4)	0.082 (7)	−0.010 (4)	−0.007 (4)	0.010 (4)
N6	0.102 (8)	0.053 (6)	0.056 (6)	−0.038 (6)	0.007 (5)	−0.019 (5)
N7	0.064 (5)	0.047 (5)	0.030 (4)	0.024 (4)	0.006 (3)	0.006 (3)
N8	0.037 (4)	0.030 (4)	0.048 (4)	0.009 (3)	0.007 (3)	0.003 (3)
N9	0.030 (4)	0.049 (4)	0.037 (4)	0.004 (3)	0.005 (3)	0.005 (3)
N10	0.091 (7)	0.053 (6)	0.047 (5)	−0.018 (5)	0.018 (5)	−0.010 (4)
N11	0.104 (8)	0.040 (5)	0.078 (7)	0.010 (5)	−0.013 (6)	0.008 (5)
N12	0.067 (6)	0.050 (6)	0.093 (8)	−0.018 (5)	0.032 (5)	−0.013 (5)
C10	0.072 (8)	0.048 (7)	0.087 (9)	−0.025 (6)	−0.029 (7)	−0.001 (6)
C11	0.083 (9)	0.055 (8)	0.127 (13)	−0.015 (7)	0.023 (8)	−0.051 (8)
N13	0.031 (4)	0.034 (4)	0.039 (4)	0.001 (3)	−0.005 (3)	−0.003 (3)
N101	0.053 (5)	0.062 (6)	0.054 (5)	−0.006 (5)	0.011 (4)	−0.003 (4)
C1	0.032 (4)	0.050 (6)	0.046 (5)	0.000 (4)	0.002 (4)	0.018 (4)
C2	0.051 (5)	0.038 (5)	0.048 (5)	0.009 (4)	0.022 (4)	0.012 (4)
C3	0.060 (6)	0.041 (5)	0.047 (6)	0.009 (5)	−0.011 (5)	−0.020 (4)
C7	0.038 (5)	0.045 (6)	0.075 (7)	−0.010 (4)	0.009 (5)	0.010 (5)
C8	0.082 (7)	0.051 (6)	0.037 (5)	−0.030 (6)	0.008 (5)	−0.015 (5)
C9	0.052 (6)	0.030 (5)	0.097 (9)	−0.002 (5)	−0.014 (6)	0.000 (5)
N5	0.062 (6)	0.027 (5)	0.111 (9)	0.005 (4)	−0.012 (5)	−0.007 (5)
C4	0.051 (5)	0.051 (6)	0.036 (5)	0.013 (5)	−0.004 (4)	−0.003 (4)
C5	0.055 (6)	0.062 (7)	0.050 (6)	0.026 (5)	0.018 (5)	−0.003 (5)
C6	0.058 (6)	0.033 (5)	0.089 (9)	0.001 (5)	0.005 (6)	0.001 (5)
C12	0.081 (8)	0.021 (5)	0.083 (8)	−0.016 (5)	−0.001 (6)	0.007 (5)
C13	0.060 (6)	0.040 (5)	0.030 (5)	0.019 (4)	0.013 (4)	−0.001 (4)
C14	0.040 (5)	0.037 (5)	0.037 (5)	0.001 (4)	0.008 (4)	−0.001 (4)
C15	0.031 (4)	0.039 (5)	0.034 (5)	0.001 (4)	0.002 (3)	−0.005 (4)
C16	0.055 (6)	0.046 (6)	0.048 (6)	0.016 (5)	0.019 (4)	0.022 (5)
C17	0.038 (5)	0.053 (6)	0.047 (6)	0.007 (4)	−0.014 (4)	0.001 (5)
C18	0.042 (5)	0.031 (5)	0.052 (6)	0.009 (4)	0.013 (4)	0.006 (4)
C19	0.091 (8)	0.057 (7)	0.036 (5)	−0.030 (6)	0.016 (5)	−0.008 (5)
C20	0.102 (9)	0.043 (6)	0.064 (8)	0.016 (6)	−0.030 (7)	0.003 (5)
C21	0.056 (6)	0.041 (6)	0.122 (11)	−0.014 (5)	0.044 (7)	−0.008 (6)
C22	0.076 (8)	0.043 (6)	0.062 (7)	−0.011 (6)	−0.018 (6)	0.008 (5)
C23	0.072 (8)	0.067 (8)	0.091 (10)	−0.012 (7)	0.029 (7)	−0.038 (7)
C24	0.171 (15)	0.040 (7)	0.054 (7)	−0.015 (8)	0.032 (8)	0.019 (5)
C25	0.018 (3)	0.029 (4)	0.022 (4)	−0.005 (3)	0.007 (3)	0.005 (3)
C26	0.057 (6)	0.044 (5)	0.031 (5)	−0.013 (5)	−0.003 (4)	−0.005 (4)
C27	0.048 (5)	0.083 (8)	0.027 (5)	0.001 (5)	0.007 (4)	−0.006 (5)
C28	0.075 (7)	0.054 (6)	0.029 (5)	−0.016 (6)	0.002 (5)	0.014 (4)
C29	0.044 (5)	0.052 (6)	0.033 (5)	0.010 (5)	−0.004 (4)	0.003 (4)
C30	0.035 (5)	0.083 (8)	0.027 (5)	−0.009 (5)	−0.006 (4)	0.002 (5)
C31	0.049 (6)	0.093 (9)	0.038 (6)	−0.004 (6)	−0.001 (4)	−0.023 (6)
C32	0.084 (8)	0.061 (7)	0.040 (6)	−0.020 (7)	0.008 (5)	−0.023 (5)
C33	0.080 (8)	0.090 (9)	0.027 (5)	0.048 (7)	−0.005 (5)	−0.018 (5)
C34	0.078 (8)	0.128 (12)	0.014 (5)	−0.028 (8)	0.002 (5)	0.008 (6)
C35	0.076 (8)	0.069 (8)	0.033 (5)	0.011 (6)	−0.022 (5)	−0.010 (5)
O1M	0.056 (4)	0.038 (4)	0.066 (5)	−0.001 (3)	0.004 (3)	−0.006 (3)
C1EB	0.067 (7)	0.067 (7)	0.093 (9)	−0.012 (6)	0.017 (6)	−0.002 (6)
C2E	0.067 (7)	0.067 (7)	0.093 (9)	−0.012 (6)	0.017 (6)	−0.002 (6)
C1EA	0.067 (7)	0.067 (7)	0.093 (9)	−0.012 (6)	0.017 (6)	−0.002 (6)

### Geometric parameters (Å, º)

**Table:**

Re1—N101	1.720 (10)	N3—C2	1.476 (11)
Re1—O1M	2.147 (6)	N3—C4	1.491 (12)
Re1—Br2	2.5085 (10)	N4—C10	1.435 (15)
Re1—Br4	2.5200 (10)	N4—C7	1.465 (13)
Re1—Br1	2.5206 (10)	N4—C12	1.466 (12)
Re1—Br3	2.5245 (10)	N6—C10	1.475 (16)
Ru1—C25	2.008 (7)	N6—C11	1.478 (17)
Ru1—C28	2.212 (9)	N6—C8	1.479 (12)
Ru1—C27	2.214 (9)	N7—C16	1.437 (12)
Ru1—C29	2.235 (8)	N7—C17	1.457 (12)
Ru1—P2	2.243 (2)	N7—C13	1.491 (11)
Ru1—C30	2.258 (8)	N8—C16	1.475 (11)
Ru1—C26	2.261 (8)	N8—C18	1.480 (11)
Ru1—P1	2.281 (2)	N8—C14	1.482 (10)
Ru2—N13	2.030 (8)	N9—C17	1.454 (12)
Ru2—C33	2.198 (9)	N9—C18	1.454 (11)
Ru2—C34	2.202 (9)	N9—C15	1.458 (11)
Ru2—C32	2.229 (9)	N10—C23	1.437 (16)
Ru2—C35	2.245 (9)	N10—C22	1.440 (14)
Ru2—C31	2.255 (9)	N10—C19	1.476 (13)
Ru2—P3	2.268 (2)	N11—C23	1.446 (16)
Ru2—P4	2.277 (2)	N11—C24	1.453 (17)
P1—C1	1.840 (8)	N11—C20	1.482 (14)
P1—C3	1.846 (9)	N12—C22	1.433 (14)
P1—C2	1.850 (8)	N12—C24	1.462 (16)
P2—C7	1.827 (9)	N12—C21	1.498 (13)
P2—C8	1.838 (9)	C11—N5	1.444 (17)
P2—C9	1.846 (10)	N13—C25	1.142 (10)
P3—C13	1.841 (8)	C9—N5	1.473 (13)
P3—C15	1.843 (8)	N5—C12	1.464 (14)
P3—C14	1.853 (9)	C26—C30	1.388 (14)
P4—C20	1.831 (10)	C26—C27	1.441 (14)
P4—C21	1.834 (10)	C27—C28	1.410 (15)
P4—C19	1.847 (9)	C28—C29	1.417 (14)
O101—N101	1.183 (11)	C29—C30	1.409 (14)
N1—C5	1.447 (12)	C31—C32	1.357 (16)
N1—C1	1.456 (11)	C31—C35	1.411 (16)
N1—C4	1.458 (12)	C32—C33	1.387 (16)
N2—C6	1.434 (14)	C33—C34	1.410 (17)
N2—C3	1.451 (12)	C34—C35	1.456 (16)
N2—C5	1.470 (13)	O1M—C1EB	1.471 (13)
N3—C6	1.473 (13)	C1EB—C2E	1.43 (3)
			
N101—Re1—O1M	178.9 (3)	C5—N1—C1	112.1 (7)
N101—Re1—Br2	94.1 (3)	C5—N1—C4	108.7 (8)
O1M—Re1—Br2	85.44 (19)	C1—N1—C4	111.3 (7)
N101—Re1—Br4	94.1 (3)	C6—N2—C3	111.5 (8)
O1M—Re1—Br4	86.33 (19)	C6—N2—C5	108.7 (8)
Br2—Re1—Br4	171.77 (4)	C3—N2—C5	110.8 (8)
N101—Re1—Br1	93.0 (3)	C6—N3—C2	110.6 (8)
O1M—Re1—Br1	85.96 (18)	C6—N3—C4	107.7 (8)
Br2—Re1—Br1	89.58 (4)	C2—N3—C4	110.6 (7)
Br4—Re1—Br1	90.10 (4)	C10—N4—C7	112.1 (8)
N101—Re1—Br3	93.0 (3)	C10—N4—C12	109.5 (10)
O1M—Re1—Br3	87.99 (18)	C7—N4—C12	110.8 (8)
Br2—Re1—Br3	89.45 (4)	C10—N6—C11	107.6 (9)
Br4—Re1—Br3	90.01 (4)	C10—N6—C8	111.2 (9)
Br1—Re1—Br3	173.93 (4)	C11—N6—C8	110.6 (9)
C25—Ru1—C28	142.5 (4)	C16—N7—C17	109.7 (7)
C25—Ru1—C27	154.2 (3)	C16—N7—C13	112.1 (7)
C28—Ru1—C27	37.2 (4)	C17—N7—C13	109.3 (7)
C25—Ru1—C29	106.9 (3)	C16—N8—C18	107.6 (7)
C28—Ru1—C29	37.1 (4)	C16—N8—C14	110.3 (6)
C27—Ru1—C29	61.3 (4)	C18—N8—C14	110.3 (7)
C25—Ru1—P2	86.3 (2)	C17—N9—C18	108.8 (7)
C28—Ru1—P2	91.3 (3)	C17—N9—C15	110.8 (7)
C27—Ru1—P2	117.6 (3)	C18—N9—C15	113.2 (7)
C29—Ru1—P2	101.4 (3)	C23—N10—C22	109.8 (9)
C25—Ru1—C30	95.6 (3)	C23—N10—C19	110.3 (9)
C28—Ru1—C30	61.6 (4)	C22—N10—C19	110.3 (9)
C27—Ru1—C30	60.9 (3)	C23—N11—C24	110.5 (10)
C29—Ru1—C30	36.5 (4)	C23—N11—C20	111.6 (10)
P2—Ru1—C30	136.4 (3)	C24—N11—C20	107.9 (11)
C25—Ru1—C26	117.0 (3)	C22—N12—C24	109.1 (9)
C28—Ru1—C26	62.5 (4)	C22—N12—C21	110.1 (9)
C27—Ru1—C26	37.5 (4)	C24—N12—C21	109.4 (10)
C29—Ru1—C26	61.1 (4)	N4—C10—N6	113.8 (8)
P2—Ru1—C26	153.3 (2)	N5—C11—N6	116.1 (10)
C30—Ru1—C26	35.8 (3)	C25—N13—Ru2	176.3 (7)
C25—Ru1—P1	88.1 (2)	O101—N101—Re1	178.6 (10)
C28—Ru1—P1	129.4 (3)	N1—C1—P1	113.5 (6)
C27—Ru1—P1	98.1 (3)	N3—C2—P1	113.1 (6)
C29—Ru1—P1	157.7 (3)	N2—C3—P1	113.4 (6)
P2—Ru1—P1	95.97 (8)	N4—C7—P2	112.5 (7)
C30—Ru1—P1	127.6 (3)	N6—C8—P2	111.6 (7)
C26—Ru1—P1	97.5 (3)	N5—C9—P2	112.7 (7)
N13—Ru2—C33	145.0 (4)	C11—N5—C12	106.9 (9)
N13—Ru2—C34	151.6 (4)	C11—N5—C9	111.3 (10)
C33—Ru2—C34	37.4 (5)	C12—N5—C9	110.9 (9)
N13—Ru2—C32	109.3 (4)	N1—C4—N3	113.3 (7)
C33—Ru2—C32	36.5 (4)	N1—C5—N2	113.8 (7)
C34—Ru2—C32	60.9 (4)	N2—C6—N3	115.1 (8)
N13—Ru2—C35	113.3 (4)	N5—C12—N4	114.0 (8)
C33—Ru2—C35	62.8 (4)	N7—C13—P3	112.5 (5)
C34—Ru2—C35	38.2 (4)	N8—C14—P3	114.2 (6)
C32—Ru2—C35	60.6 (4)	N9—C15—P3	112.3 (6)
N13—Ru2—C31	94.7 (3)	N7—C16—N8	114.5 (7)
C33—Ru2—C31	60.9 (4)	N9—C17—N7	114.3 (7)
C34—Ru2—C31	61.5 (4)	N9—C18—N8	113.6 (7)
C32—Ru2—C31	35.2 (4)	N10—C19—P4	112.9 (7)
C35—Ru2—C31	36.6 (4)	N11—C20—P4	113.0 (7)
N13—Ru2—P3	86.25 (19)	N12—C21—P4	112.5 (7)
C33—Ru2—P3	94.2 (3)	N12—C22—N10	115.9 (9)
C34—Ru2—P3	121.3 (4)	N10—C23—N11	114.2 (9)
C32—Ru2—P3	102.5 (3)	N11—C24—N12	114.9 (9)
C35—Ru2—P3	156.9 (3)	N13—C25—Ru1	175.5 (7)
C31—Ru2—P3	134.9 (3)	C30—C26—C27	106.4 (9)
N13—Ru2—P4	85.8 (2)	C30—C26—Ru1	72.0 (5)
C33—Ru2—P4	128.6 (4)	C27—C26—Ru1	69.4 (5)
C34—Ru2—P4	96.7 (4)	C28—C27—C26	108.9 (9)
C32—Ru2—P4	155.8 (3)	C28—C27—Ru1	71.4 (5)
C35—Ru2—P4	96.4 (3)	C26—C27—Ru1	73.0 (5)
C31—Ru2—P4	127.9 (3)	C27—C28—C29	106.7 (9)
P3—Ru2—P4	97.13 (7)	C27—C28—Ru1	71.5 (5)
C1—P1—C3	96.6 (5)	C29—C28—Ru1	72.3 (5)
C1—P1—C2	96.5 (4)	C30—C29—C28	108.3 (9)
C3—P1—C2	97.4 (5)	C30—C29—Ru1	72.6 (5)
C1—P1—Ru1	126.3 (3)	C28—C29—Ru1	70.6 (5)
C3—P1—Ru1	114.4 (3)	C26—C30—C29	109.6 (8)
C2—P1—Ru1	119.8 (3)	C26—C30—Ru1	72.2 (5)
C7—P2—C8	99.0 (5)	C29—C30—Ru1	70.8 (5)
C7—P2—C9	96.6 (5)	C32—C31—C35	109.4 (10)
C8—P2—C9	98.5 (6)	C32—C31—Ru2	71.4 (6)
C7—P2—Ru1	122.8 (4)	C35—C31—Ru2	71.4 (5)
C8—P2—Ru1	120.6 (3)	C31—C32—C33	110.7 (11)
C9—P2—Ru1	114.4 (3)	C31—C32—Ru2	73.4 (6)
C13—P3—C15	97.8 (4)	C33—C32—Ru2	70.5 (6)
C13—P3—C14	96.6 (4)	C32—C33—C34	106.9 (10)
C15—P3—C14	96.9 (4)	C32—C33—Ru2	73.0 (6)
C13—P3—Ru2	120.6 (3)	C34—C33—Ru2	71.4 (6)
C15—P3—Ru2	124.4 (3)	C33—C34—C35	107.8 (11)
C14—P3—Ru2	115.0 (3)	C33—C34—Ru2	71.2 (6)
C20—P4—C21	97.7 (6)	C35—C34—Ru2	72.5 (6)
C20—P4—C19	97.3 (6)	C31—C35—C34	105.2 (10)
C21—P4—C19	96.7 (5)	C31—C35—Ru2	72.1 (6)
C20—P4—Ru2	113.5 (4)	C34—C35—Ru2	69.3 (5)
C21—P4—Ru2	125.0 (4)	C1EB—O1M—Re1	128.3 (6)
C19—P4—Ru2	121.1 (3)	C2E—C1EB—O1M	147.8 (14)
			
C7—N4—C10—N6	67.9 (11)	C13—N7—C16—N8	67.5 (10)
C12—N4—C10—N6	−55.4 (11)	C18—N8—C16—N7	54.6 (9)
C11—N6—C10—N4	53.3 (12)	C14—N8—C16—N7	−65.7 (10)
C8—N6—C10—N4	−68.0 (12)	C18—N9—C17—N7	−54.8 (10)
C10—N6—C11—N5	−54.7 (12)	C15—N9—C17—N7	70.3 (10)
C8—N6—C11—N5	67.0 (13)	C16—N7—C17—N9	53.9 (10)
C5—N1—C1—P1	−59.7 (9)	C13—N7—C17—N9	−69.3 (10)
C4—N1—C1—P1	62.2 (9)	C17—N9—C18—N8	56.1 (9)
C3—P1—C1—N1	48.0 (7)	C15—N9—C18—N8	−67.6 (9)
C2—P1—C1—N1	−50.2 (7)	C16—N8—C18—N9	−55.5 (9)
Ru1—P1—C1—N1	175.0 (5)	C14—N8—C18—N9	64.8 (9)
C6—N3—C2—P1	58.7 (9)	C23—N10—C19—P4	61.3 (11)
C4—N3—C2—P1	−60.6 (9)	C22—N10—C19—P4	−60.2 (11)
C1—P1—C2—N3	49.7 (8)	C20—P4—C19—N10	−48.6 (10)
C3—P1—C2—N3	−47.8 (8)	C21—P4—C19—N10	50.1 (10)
Ru1—P1—C2—N3	−171.5 (5)	Ru2—P4—C19—N10	−171.8 (7)
C6—N2—C3—P1	−60.1 (10)	C23—N11—C20—P4	−59.0 (14)
C5—N2—C3—P1	61.1 (10)	C24—N11—C20—P4	62.6 (12)
C1—P1—C3—N2	−49.3 (8)	C21—P4—C20—N11	−50.6 (11)
C2—P1—C3—N2	48.1 (8)	C19—P4—C20—N11	47.2 (11)
Ru1—P1—C3—N2	175.7 (6)	Ru2—P4—C20—N11	175.8 (9)
C10—N4—C7—P2	−59.8 (10)	C22—N12—C21—P4	60.6 (12)
C12—N4—C7—P2	62.8 (10)	C24—N12—C21—P4	−59.3 (12)
C8—P2—C7—N4	47.8 (8)	C20—P4—C21—N12	48.4 (10)
C9—P2—C7—N4	−52.0 (8)	C19—P4—C21—N12	−49.9 (10)
Ru1—P2—C7—N4	−176.6 (5)	Ru2—P4—C21—N12	174.3 (7)
C10—N6—C8—P2	59.5 (11)	C24—N12—C22—N10	52.2 (12)
C11—N6—C8—P2	−60.0 (12)	C21—N12—C22—N10	−67.9 (12)
C7—P2—C8—N6	−48.0 (9)	C23—N10—C22—N12	−53.9 (12)
C9—P2—C8—N6	50.1 (9)	C19—N10—C22—N12	67.9 (12)
Ru1—P2—C8—N6	175.1 (7)	C22—N10—C23—N11	52.8 (12)
C7—P2—C9—N5	50.9 (10)	C19—N10—C23—N11	−69.0 (12)
C8—P2—C9—N5	−49.2 (10)	C24—N11—C23—N10	−52.0 (13)
Ru1—P2—C9—N5	−178.5 (8)	C20—N11—C23—N10	68.0 (13)
N6—C11—N5—C12	55.5 (12)	C23—N11—C24—N12	50.9 (14)
N6—C11—N5—C9	−65.7 (13)	C20—N11—C24—N12	−71.3 (13)
P2—C9—N5—C11	58.3 (12)	C22—N12—C24—N11	−50.4 (13)
P2—C9—N5—C12	−60.5 (12)	C21—N12—C24—N11	70.2 (13)
C5—N1—C4—N3	56.5 (10)	C30—C26—C27—C28	−0.2 (10)
C1—N1—C4—N3	−67.4 (10)	Ru1—C26—C27—C28	62.7 (6)
C6—N3—C4—N1	−54.3 (10)	C30—C26—C27—Ru1	−63.0 (6)
C2—N3—C4—N1	66.7 (10)	C26—C27—C28—C29	0.4 (10)
C1—N1—C5—N2	66.4 (10)	Ru1—C27—C28—C29	64.2 (6)
C4—N1—C5—N2	−57.0 (10)	C26—C27—C28—Ru1	−63.8 (6)
C6—N2—C5—N1	55.9 (11)	C27—C28—C29—C30	−0.4 (10)
C3—N2—C5—N1	−67.0 (10)	Ru1—C28—C29—C30	63.2 (6)
C3—N2—C6—N3	67.6 (11)	C27—C28—C29—Ru1	−63.6 (6)
C5—N2—C6—N3	−54.8 (11)	C27—C26—C30—C29	0.0 (10)
C2—N3—C6—N2	−66.8 (11)	Ru1—C26—C30—C29	−61.3 (6)
C4—N3—C6—N2	54.2 (11)	C27—C26—C30—Ru1	61.3 (6)
C11—N5—C12—N4	−55.5 (12)	C28—C29—C30—C26	0.3 (10)
C9—N5—C12—N4	66.0 (13)	Ru1—C29—C30—C26	62.2 (6)
C10—N4—C12—N5	56.9 (11)	C28—C29—C30—Ru1	−61.9 (6)
C7—N4—C12—N5	−67.2 (12)	C35—C31—C32—C33	0.5 (12)
C16—N7—C13—P3	−61.3 (9)	Ru2—C31—C32—C33	−61.0 (7)
C17—N7—C13—P3	60.6 (9)	C35—C31—C32—Ru2	61.6 (7)
C15—P3—C13—N7	−49.0 (7)	C31—C32—C33—C34	−1.1 (11)
C14—P3—C13—N7	48.9 (7)	Ru2—C32—C33—C34	−63.8 (7)
Ru2—P3—C13—N7	173.0 (5)	C31—C32—C33—Ru2	62.8 (7)
C16—N8—C14—P3	59.5 (8)	C32—C33—C34—C35	1.2 (11)
C18—N8—C14—P3	−59.3 (8)	Ru2—C33—C34—C35	−63.7 (6)
C13—P3—C14—N8	−49.6 (7)	C32—C33—C34—Ru2	64.9 (7)
C15—P3—C14—N8	49.1 (6)	C32—C31—C35—C34	0.2 (11)
Ru2—P3—C14—N8	−177.7 (5)	Ru2—C31—C35—C34	61.8 (6)
C17—N9—C15—P3	−61.0 (8)	C32—C31—C35—Ru2	−61.6 (7)
C18—N9—C15—P3	61.6 (8)	C33—C34—C35—C31	−0.8 (10)
C13—P3—C15—N9	49.0 (7)	Ru2—C34—C35—C31	−63.7 (7)
C14—P3—C15—N9	−48.7 (6)	C33—C34—C35—Ru2	62.8 (6)
Ru2—P3—C15—N9	−175.4 (4)	Re1—O1M—C1EB—C2E	−144 (3)
C17—N7—C16—N8	−54.2 (10)		

### Hydrogen-bond geometry (Å, º)

**Table:**

*D*—H···*A*	*D*—H	H···*A*	*D*···*A*	*D*—H···*A*
C10—H10*A*···Br3^i^	0.97	3.12	3.944 (12)	143
C10—H10*B*···Br2	0.97	2.83	3.709 (10)	150
C1—H1*B*···Br4^ii^	0.97	3.03	3.967 (9)	163
C7—H7*B*···N9^iii^	0.97	2.59	3.309 (11)	131
C8—H8*A*···Br3^i^	0.97	2.89	3.772 (12)	151
C4—H4*B*···Br3^i^	0.97	3.10	4.062 (10)	169
C5—H5*A*···Br1^ii^	0.97	3.10	3.918 (10)	143
C18—H18*A*···N4^iv^	0.97	2.53	3.208 (11)	127
C18—H18*B*···Br2^v^	0.97	2.92	3.858 (9)	163
C19—H19*B*···Br1^vi^	0.97	3.09	3.938 (11)	147
C22—H22*B*···Br1^vi^	0.97	3.00	3.861 (10)	148
C23—H23*A*···Br4^vii^	0.97	3.10	4.007 (12)	156
C24—H24*A*···Br3^vii^	0.97	2.98	3.799 (11)	143
O1*M*—H1*m*···N8^iii^	0.85	1.88	2.709 (9)	166
C1*EB*—H101···Br3	0.97	2.80	3.527 (13)	132
C2*E*—H2*e*3···N6^i^	0.96	2.36	3.15 (3)	140

Symmetry codes: (i) −*x*, −*y*, −*z*; (ii) −*x*, *y*+1/2, −*z*+1/2; (iii) *x*−1, *y*, *z*; (iv) *x*+1, *y*, *z*; (v) −*x*+1, −*y*, −*z*; (vi) −*x*+1, *y*+1/2, −*z*+1/2; (vii) *x*+1, *y*+1, *z*.
