# Supplementary figures and images for: Crystal structure and magnetic study of the complex salt [RuCp(PTA)2–μ-CN-1κC:2κN–RuCp(PTA)2][Re(NO)Br4(EtOH)0.5(MeOH)0.5]
Source: Acta Crystallogr E Crystallogr Commun. 2021 Jun 30;77(Pt 7):749–54. doi: 10.1107/S2056989021006381 (PMC8382050; doi:10.1107/S2056989021006381)

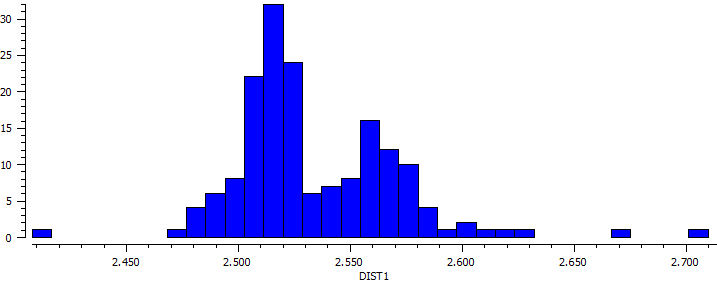

Supplement: Supplementary file 3 [file e-77-00749-sup3.png]

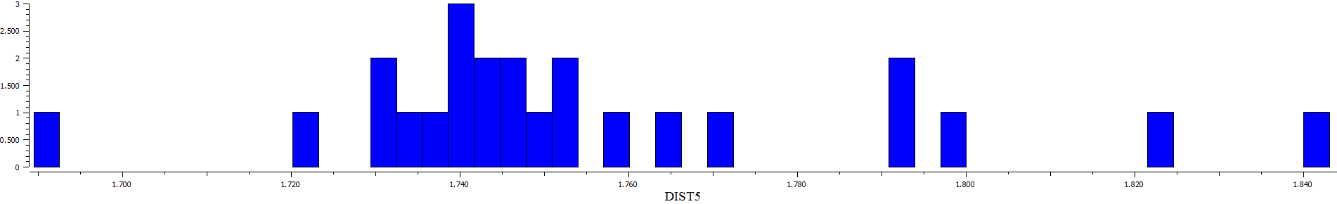

Supplement: Supplementary file 4 [file e-77-00749-sup4.png]

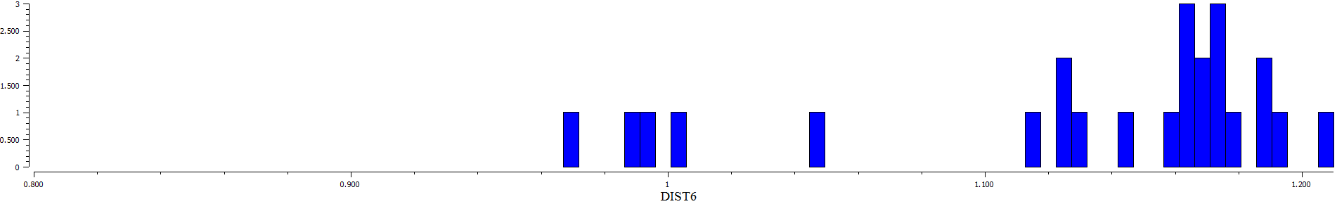

Supplement: Supplementary file 5 [file e-77-00749-sup5.png]

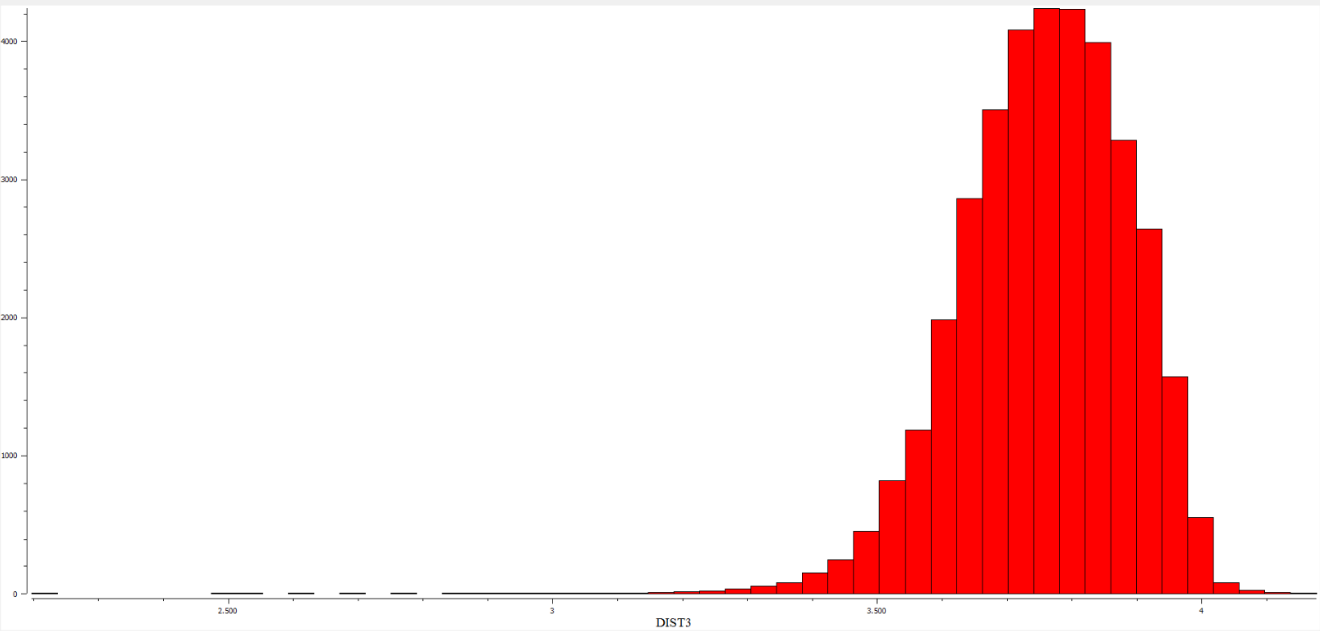

Supplement: Supplementary file 6 [file e-77-00749-sup6.png]

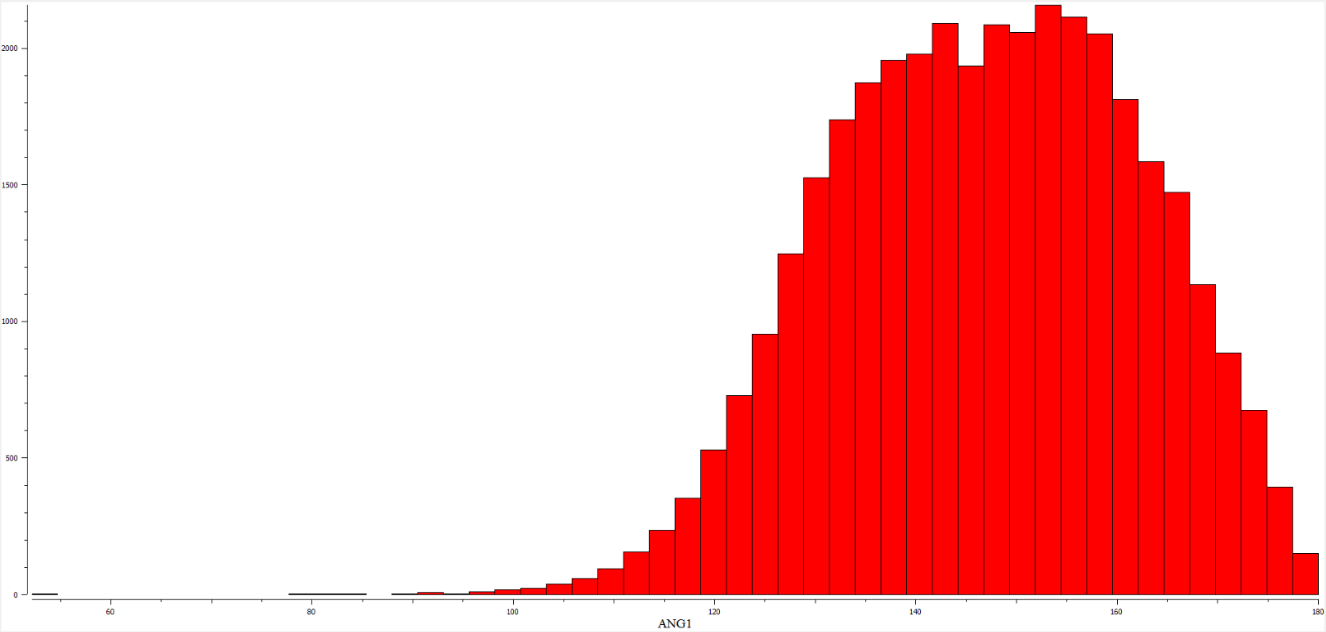

Supplement: Supplementary file 7 [file e-77-00749-sup7.png]
